# Dosimetric comparison of VMAT with integrated skin flash to 3D field‐in‐field tangents for left breast irradiation

**DOI:** 10.1002/acm2.12527

**Published:** 2019-01-17

**Authors:** Jonathan Bogue, Jui Wan, Robert S. Lavey, E. Ishmael Parsai

**Affiliations:** ^1^ Department of Radiation Oncology University of Toledo Medical Center Toledo OH USA; ^2^ Maurer Family Cancer Center Bowling Green OH USA

**Keywords:** breast cancer, organ dose, VMAT, optimization, tangential

## Abstract

Volumetric modulated arc therapy (VMAT) has been implemented for left breast irradiation to reduce prescription dose to the heart and improve dose homogeneity across the targeted breast. Our in‐house method requires application of a bolus during the optimization process with a target outside of the body, then removing the bolus during the final calculation in order to incorporate skin flash in VMAT plans. To quantify the dosimetric trade‐offs between traditional 3D field‐in‐field tangents and VMAT with integrated skin flash for these patients, we compared nine consecutive patients who recently received radiation to their entire left breast but not their regional lymphatics. Tangent plans used non‐divergent tangents of mixed energies and VMAT plans utilized four 6 MV arcs of roughly 260°. Mean dose to the heart, contralateral lung, and contralateral breast and their volume receiving 5%, 10%, and 20% of the prescription dose were higher in all nine VMAT plans than in the static tangential beam plans. For all critical structures, the mean VMAT DVH was higher in the low‐dose region and crossed the 3D field‐in‐field DVH between 23.13% and 34.18% of the prescription dose (984.75‐1454.70 cGy). However, the volume of the contralateral breast and heart receiving the prescription dose was slightly lower in the VMAT plans, but not statistically significant. VMAT provided superior homogeneity, with a mean homogeneity index of 9.41 ± 1.64 compared to 11.05 ± 1.82 for 3D tangents. Results indicate that VMAT spares the heart, contralateral lung, and contralateral breast from prescription dose at the cost of increasing their mean and low‐dose volume and delivers a more homogenous dose distribution to the breast. For these reasons, VMAT is selectively applied at the request of the physician for left breast radiation without respiratory gating to spare the heart from prescription dose in cases of poor anatomical geometry.

## 1 INTRODUCTION

The application of VMAT to left breast irradiation has raised concern about potential cardiac toxicity. Respiratory gating techniques such as voluntary deep inspiration breath‐hold (DIBH), which has been shown to protect the heart by displacing it from the chest wall, are not available at many institutions.^1^, ^2^ VMAT and intensity‐modulated radiotherapy (IMRT) with built‐in skin flash may decrease the heart dose but increase low‐dose exposure of nearby healthy tissues compared with three‐dimensional (3D) field‐in‐field tangents.^3^, ^4^, ^5^, ^6^, ^7^, ^8^, ^9^ Selected cases have been presented where our radiation oncologists have requested VMAT to spare large volumes of the heart from receiving prescription dose. While a proportional increase in ischemic heart disease has been linked with the heart mean dose, it is not known if it is better for less of the heart to receive a high dose or more of the heart to receive a low dose.^10^, ^11^, ^12^ The use of VMAT with integrated skin flash has been clinically accepted by our physicians. This study quantifies the dosimetric trade‐offs and crossover points between low‐ and high‐dose volumes for left breast cancer patients treated with the VMAT with the integrated skin flash planning method compared to results obtained using conventional 3D planning techniques.

## 2 MATERIALS AND METHODS

Left breast radiotherapy plans were compared for traditional 3D field‐in‐field tangent plans and four partial arcs VMAT using the Eclipse treatment planning system (Varian Medical Systems, Palo Alto, CA).

### 2.A Patient selection

The study included nine consecutive left breast cancer patients without axillary lymph nodes involvement who were treated in the supine position without respiratory gating or voluntary breath hold by a single radiation oncologist. The physician requested eight patients be planned and treated using VMAT and one be planned and treated using 3D field‐in‐field tangents. Patients were prescribed 4256 cGy to their entire left breast in 16 fractions of 266 cGy each. Patient data sets were transferred to MIM Maestro (MIM Software Inc, Cleveland, OH) for image registration if PET is requested and organs at risk (OAR) delineation. The physician specified the planned target volume and approved the OAR contours.

### 2.B Treatment planning

**Figure 1:**
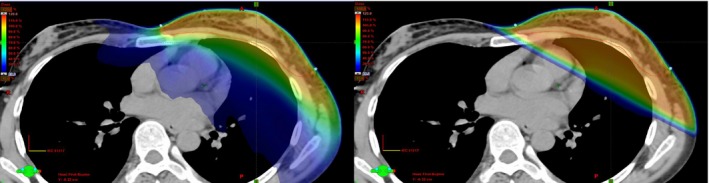
Left panel is a volumetric modulated arc therapy plan optimized to meet the constraints set by the physician, and the right panel is a three‐dimensional field‐in‐field plan with tangents placed to encompass the left breast while maximizing sparing of the ipsilateral lung, heart, and contralateral breast.

Two plans were created for each patient utilizing field‐in‐field tangents and VMAT with included flash. Figure 1 shows the typical isodose distributions achieved using each method for the same patient. The VMAT plans utilized four 6 MV partial arcs rotating from gantry 180°‐300°, with the endpoint varying ±10° depending on patient anatomy. The collimator was set to ±30° to avoid the overlapping tongue and groove effect and the X‐jaw positions were defined to prevent the maximum X‐jaw motion from exceeding 14.5 cm to allow full MLC modulation of the field. Maximum leaf travel and the field width necessitated the use of four arcs to allow sufficient modulation of the entire field width. To accommodate skin flash, plans were optimized with 1 cm of bolus of 0.6 g/cm^3^ and an optimization PTV that was expanded 8 mm anteriorly to simulate the chest motion.^13^ All optimizations were performed with the bolus linked to the fields. The bolus was removed on the final dose calculation to simulate treatment conditions. After the final dose calculation, plans were normalized to cover 95% of the physician's original target volume with 100% prescription dose.

Three‐dimensional field‐in‐field plans utilized non‐divergent tangents of mixed energies to optimize homogeneity. Tangent angles were chosen to minimize dose to the heart, ipsilateral lung, and contralateral breast while still covering the target volume. MLC‐defined control points were created to minimize hot spots and improve homogeneity.

**Figure 2:**
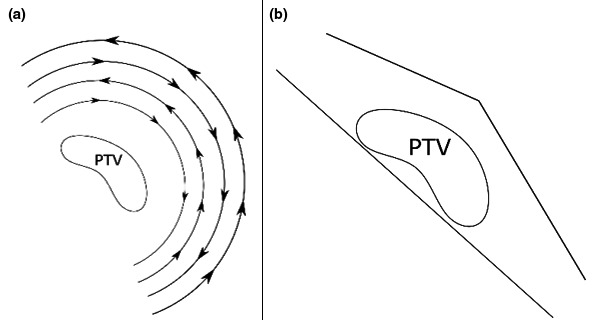
Beam arrangement for (a) volumetric modulated arc therapy using four arcs and (b) three‐dimensional field‐in‐field using non‐divergent tangents with flash.

Figure 2 illustrates the VMAT and 3D field arrangements. A 2 mm dose grid was applied to all cases and the AcurosXB dose calculation was used (Varian Medical Systems, Palo Alto, CA).

Pre‐treatment imaging was performed as requested by the physician, with the routine CBCT to confirm that the breast was within the optimized treatment volume and swelling or respiratory motion had not exceeded the planned motion.

### 2.C Analysis

To quantify both the expected increase in low‐dose scatter by VMAT and volume of prescription dose to nearby critical structures by 3D field‐in‐field plans, the heart, contralateral lung, and contralateral breast were evaluated based on the mean dose and relative volume receiving 5% (V5), 10% (V10), 20% (V20), 30% (V30), 40% (V40), and 100% (V100) of the prescription dose. Homogeneity index (HI) was calculated to evaluate dose uniformity within the target using:

(1) $$HI=\frac{D_{2}-D_{98}}{D_{p}}\times 100$$

where *D*_2_ = minimum dose to 2% of the target volume, *D*_98_ = minimum dose to 98% of the target volume, and *D*_p_ = prescribed dose.^14^ Using this index, a lower HI value indicates a more homogenous dose distribution.

## 3 RESULTS

Table 1 shows the minimum, maximum, mean, and standard deviations for all criteria evaluated for both VMAT and 3D field‐in‐field delivery techniques. The VMAT plan produced a higher overall mean dose and V5, V10, and V20 for the heart, contralateral lung, and contralateral breast in every patient. The volume of the contralateral breast and heart receiving the prescription dose was slightly lower in the VMAT plans, but not statistically significant. Treatment method did not significantly affect the volume of contralateral lung receiving the prescription dose. VMAT provided superior homogeneity, with a mean homogeneity index of 9.41 ± 1.64 for VMAT compared to 11.05 ± 1.82 for 3D field‐in‐field tangents.

**Table 1:** Results for plan evaluation criteria for each organ at risk. Values reported as 0.000 are less than 0.001

Structures	Minimum	3D field‐in‐field		SD	Minimum	VMAT		SD	*P* Value
		Maximum	Mean			Maximum	Mean		
Heart									
Mean (cGy)	49.2	910.1	399.53	260.14	192.9	961.5	747.53	249.44	0.011
V5 (%)	1.85	34.53	22.66	12.12	32.06	100	90.65	22.55	0
V10 (%)	0.04	25.09	11.6	8.42	10.33	96.75	77.74	29.36	0
V20 (%)	0	21.75	8.75	7.13	0.35	54.55	33.36	17.54	0.002
V30 (%)	0	20.21	7.74	6.58	0	17.17	10.48	5.93	0.245
V40 (%)	0	19.06	7.06	6.19	0	7.47	3.77	2.54	0.291
V100 (%)	0	12.06	3.11	4	0	0	0	0	0.066
Contralateral lung									
Mean (cGy)	17.7	51.5	31.93	12.01	193.7	436.5	299.82	70.13	0.000
V5 (%)	0	2.76	0.89	1.05	29.58	98.67	66.78	20.12	0.000
V10 (%)	0	0.95	0.16	0.31	7.31	39.35	17.62	9.85	0.001
V20 (%)	0	0.36	0.05	0.12	0.09	4.48	1.38	1.73	0.05
V30 (%)	0	0.21	0.02	0.07	0	0.77	0.1	0.25	0.424
V40 (%)	0	0.14	0.02	0.05	0	0.19	0.02	0.06	0.838
V100 (%)	0	0	0	0	0	0	0	0	*
Ipsilateral lung									
Mean (cGy)	188.79	871.42	573.71	195.78	234.08	891.42	674.58	192.37	0.288
V5 (%)	19.52	42.50	33.26	7.59	8.3	99.95	82.87	29.48	0.001
V10 (%)	11.99	31.42	23.66	6.3	7.93	95.18	67.1	28.26	0.003
V20 (%)	6.3	24.42	17.15	5.57	7.12	36.61	25.39	9.83	0.048
V30 (%)	3.88	22.19	14.57	5.24	4.19	20.8	13.09	4.4	0.532
V40 (%)	2.69	20.86	13.13	5.18	0.88	14.36	8.26	3.98	0.041
V100 (%)	0	10.45	2.44	3.28	0	0.17	0.03	0.06	0.105
Contralateral breast									
Mean (cGy)	94.1	799.1	305.76	282.21	248.9	738.4	422.87	159.11	0.298
V5 (%)	5.75	28.95	13.42	9.04	33.36	85.95	67.49	17.52	0
V10 (%)	2.48	21.22	8.26	7.72	11.88	51.37	28.2	13.94	0.003
V20 (%)	1.64	19.21	6.86	7.19	2.97	24.89	9.6	8.04	0.458
V30 (%)	0.93	18.15	6.18	6.83	0.64	16.2	4.79	5.48	0.641
V40 (%)	0.5	17.33	5.68	6.54	0.04	11.13	2.73	3.88	0.266
V100 (%)	0	9.9	2.86	3.99	0	0.06	0.01	0.02	0.064

**Figure 3:**
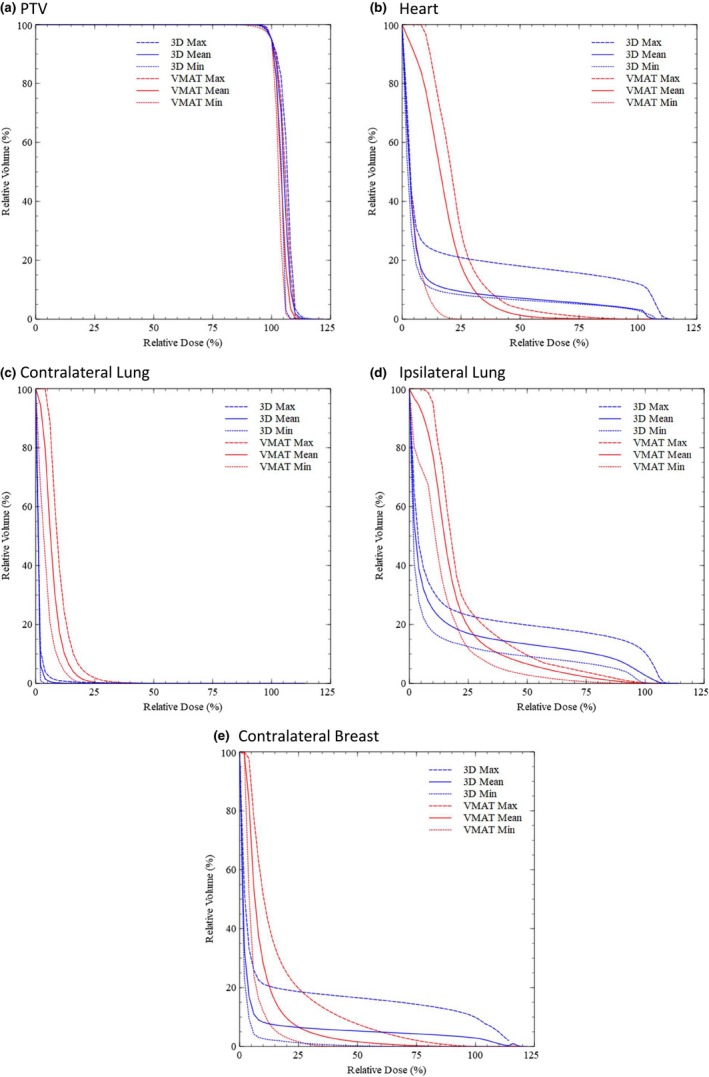
The maximum, minimum, and mean DVH curves for (a) PTV, (b) heart, (c) contralateral lung, (d) ipsilateral lung, and (e) contralateral breast.

Figure 3 shows the mean, minimum, and maximum DVH curves for each evaluated structure with the VMAT and 3D field‐in‐field plans. The intersection point for the mean DVH curves was calculated for each structure.

### 3.A PTV coverage and homogeneity

All plans were normalized to cover 95% of the PTV with 100% prescription dose. The average *D*_2_ was 4583.15 ± 55.52 cGy for VMAT and 4649.59 ± 70.40 cGy for 3D field‐in‐field tangents. VMAT also produced a better *D*_98_ of 4182.71 ± 22.27 cGy compared to 4179.32 ± 12.79 cGy for 3D field‐in‐field tangents. Therefore, the HI was smaller for VMAT, meaning it produced a more homogeneous plan with a value of 9.41 ± 1.64 vs 11.05 ± 1.82 for 3D field‐in‐field.

### 3.B Heart

VMAT decreased the V100 and V40 volumes as compared to field‐in‐field, but at the cost of increasing the V5, V10, V20, V30, and mean dose. The mean DVH curve for VMAT was higher than the field‐in‐field curve for doses up to 34.18% of the prescription dose (1454.70 cGy). At higher doses, the mean field‐in‐field curve was higher.

### 3.C Contralateral lung

Mean dose and V5, V10, and V20 were higher with VMAT plans than with field‐in‐field for the contralateral lung. The mean DVH curve for VMAT was higher than the field‐in‐field curve for doses up to 27.15% of the prescription dose (1155.50 cGy). At higher doses, the mean field‐in‐field curve was higher.

### 3.D Ipsilateral lung

Ipsilateral lung mean dose, V5, V10, and V20 were higher, but V30, V40, and V100 were lower with VMAT plans. The mean DVH curve for VMAT was higher than the field‐in‐field curve for doses up to 28.78% of the prescription dose (1012.08 cGy). At higher doses, the mean field‐in‐field curve was higher.

### 3.E Contralateral breast

The average mean dose and V5, V10, and V20 were higher with VMAT compared to field‐in‐field for the contralateral breast. The mean DVH curve for VMAT was higher than the field‐in‐field curve for doses up to 23.13% of the prescription dose (984.75 cGy). Above this dose, the mean field‐in‐field curve was higher.

## 4 DISCUSSION

Three‐dimensional field‐in‐field produces lower mean doses and V5, V10, and V20 for the heart, contralateral lung, and contralateral breast than VMAT due to the low‐dose spread of VMAT. 3D field‐in‐field plans did, however, show a slight increase in relative volume receiving the prescription dose for the contralateral breast and heart but was not statistically significant. There was no difference between the two methods in the contralateral lung. VMAT produces superior homogeneity, with a mean value of 9.41 ± 1.64 compared to 11.05 ± 1.82 for field‐in‐field. VMAT also resulted in a lower *D*_2_ to the targeted breast, which may result in less desquamation and fibrosis, and to the contralateral breast, which may result in fewer radiation‐induced breast cancers. Hot spots were minimized by the planner as low as achievable, being limited by the superficial skin and target volume coverage deemed acceptable by the physician.

The crossover point for the mean DVH curves provides an estimate for the dose level above which VMAT is beneficial. The mean DVH curves for heart, contralateral lung, ipsilateral lung, and contralateral breast were all higher in the low‐dose region for VMAT, crossing the mean 3D field‐in‐field curve between 23.13% and 34.18% of the prescription dose (984.75‐1454.70 cGy). Therefore, if the clinician is primarily interested in the mean dose to OAR or the OAR volume receiving less than approximately 30% of the prescription, the 3D field‐in‐field tangent treatment method should be used. However, if the clinician is more concerned with the maximum dose, dose homogeneity to the target, and volumes of the OARs receiving above 30% of the prescribed dose, the VMAT treatment method will be preferred.

Given the smaller sample size of nine patients, the *P* value was computed to evaluate the significance of the results. As seen in Table 1, the significance of the results varies for parameter evaluated. However, the mean dose and low‐dose volume of the heart and the low‐dose volume of both ipsilateral and contralateral lung were statistically significant. The trends presented by the maximum, minimum, and mean data were observed in all cases on a head‐to‐head plan analysis. All VMAT plans utilized the same treatment planning technique, different from other documented VMAT breast planning methods.^3^, ^5^ Despite the different planning technique, our study produces comparable results while further quantifying the dosimetric trade‐offs between VMAT and 3D field‐in‐field tangents.^5^

## 5 CONCLUSIONS

VMAT radiotherapy is used for treatment of left breast in numerous centers. While respiratory gating and the use of voluntary DIBH have been shown to reduce heart dose, it is not yet available in many centers. This study evaluated a method of using VMAT incorporating skin flash that can easily be implemented in the Eclipse TPS by applying bolus to the optimization process. Among patients whose entire left breast was treated in the supine position with free‐breathing, VMAT spared the heart, contralateral lung, and contralateral breast from prescription dose, delivered a more homogenous dose distribution to the targeted breast, and decreased the maximum breast dose compared to an optimized 3D field‐in‐field tangent treatment technique. However, VMAT produced higher mean and low‐dose volumes to the organs at risk. The crossover value of the mean DVH curves occurred at 23.13%‐34.18% of the prescription dose. In comparison with a 3D‐conformal technique, VMAT improves dose homogeneity and high‐dose exposure of OARs at the expense of mean OAR dose and OAR volume receiving less than approximately 30% of the prescription dose.

## CONFLICT OF INTERESTS

None to report.

## References

[1] Bruzzaniti V , Abate A , Pinnarò P , et al. Dosimetric and clinical advantages of deep inspiration breath‐hold (DIBH) during radiotherapy of breast cancer. J Exp Clin Cancer Res. 2013;32:88. 24423396 10.1186/1756-9966-32-88 PMC3826503

[2] Reardon KA , Read PW , Morris MM , Reardon MA , Geesey C , Wijesooriya K . A comparative analysis of 3D conformal deep inspiratory–breath hold and free‐breathing intensity‐modulated radiation therapy for left‐sided breast cancer. Med Dosim. 2013;38:190–195. 23453454 10.1016/j.meddos.2013.01.002

[3] Jin G‐H , Chen L‐X , Deng X‐W , Liu X‐W , Huang Y , Huang X‐B . A comparative dosimetric study for treating left‐sided breast cancer for small breast size using five different radiotherapy techniques: conventional tangential field, filed‐in‐filed, tangential‐IMRT, multi‐beam IMRT and VMAT. Radiat Oncol. 2013;8:89. 23587298 10.1186/1748-717X-8-89 PMC3648459

[4] Qiu J‐J , Chang Z , Horton JK , Wu Q‐RJ , Yoo S , Yin F‐F . Dosimetric comparison of 3D conformal, IMRT, and V‐MAT techniques for accelerated partial‐breast irradiation (APBI). Med Dosim. 2014;39:152–158. 24480375 10.1016/j.meddos.2013.12.001

[5] Jeulink M , Dahele M , Meijnen P , Slotman BJ , Verbakel WF . Is there a preferred IMRT technique for left‐breast irradiation? J Appl Clin Med Phys. 2015;16:197–205. 10.1120/jacmp.v16i3.5266 PMC5690145 26103488

[6] Qi XS , Liu TX , Liu AK , et al. Left‐sided breast cancer irradiation using rotational and fixed‐field radiotherapy. Med Dosim. 2014;39:227–234. 24857697 10.1016/j.meddos.2014.02.005

[7] Lee B , Lee S , Sung J , Yoon M . Radiotherapy‐induced secondary cancer risk for breast cancer: 3D conformal therapy versus IMRT versus VMAT. J Radiol Prot. 2014;34:325–331. 24705154 10.1088/0952-4746/34/2/325

[8] Haertl PM , Pohl F , Weidner K , Groeger C , Koelbl O , Dobler B . Treatment of left sided breast cancer for a patient with funnel chest: volumetric‐modulated arc therapy vs. 3D‐CRT and intensity‐modulated radiotherapy. Med Dosim. 2013;38:1–4. 22727550 10.1016/j.meddos.2012.04.003

[9] Adam D , Suditu MD , Popa R , Ciocaltei V . Volumetric‐modulated arc therapy vs. 3D‐conformal radiotherapy for breast cancer. Rom Rep Phys. 2015;67:978–986.

[10] Darby SC , Ewertz M , McGale P , et al. Risk of ischemic heart disease in women after radiotherapy for breast cancer. N Engl J Med. 2013;368:987–998. 23484825 10.1056/NEJMoa1209825

[11] Little MP , Kleinerman RA , Stovall M , Smith SA , Mabuchi K . Analysis of dose response for circulatory disease after radiotherapy for benign disease. Int J Radiat Oncol Biol Phys. 2012;84:1101–1109. 22494591 10.1016/j.ijrobp.2012.01.053 PMC3396750

[12] Taylor CW , Nisbet A , McGale P , Darby SC . Cardiac exposures in breast cancer radiotherapy: 1950s–1990s. Int J Radiat Oncol. 2007;69:1484–1495. 10.1016/j.ijrobp.2007.05.034 18035211

[13] Nicolini G , Fogliata A , Clivio A , Vanetti E , Cozzi L . Planning strategies in volumetric modulated arc therapy for breast: planning strategies in VMAT for breast. Med Phys. 2011;38:4025–4031.21859000

[14] Kataria T , Sharma K , Subramani V , Karrthick K , Bisht S . Homogeneity index: an objective tool for assessment of conformal radiation treatments. J Med Phys. 2012;37:207. 23293452 10.4103/0971-6203.103606 PMC3532749

